# Disseminated Tumor Cells in Bone Marrow of Gastric Cancer Patients: Correlation with Tumor Hypoxia and Clinical Relevance

**DOI:** 10.1155/2014/582140

**Published:** 2014-02-11

**Authors:** Larissa Bubnovskaya, Antonina Kovelskaya, Lilya Gumenyuk, Irina Ganusevich, Lesya Mamontova, Victor Mikhailenko, Dmitry Osinsky, Sergej Merentsev, Sergej Osinsky

**Affiliations:** ^1^R.E. Kavetsky Institute of Experimental Pathology, Oncology and Radiobiology, National Academy of Sciences of Ukraine, Vasilkovskaya Street 45, Kiev 03022, Ukraine; ^2^City Clinical Oncological Center, Verchovynna Street 69, Kiev 03115, Ukraine

## Abstract

*Aim. *The evaluation of the clinical relevance of disseminated tumor cells (DTCs) in bone marrow (BM) of patients with gastric cancer (GC) and their association with primary tumor hypoxia. *Patients and Methods. *89 resected specimens were used. DTCs were detected using immunocytochemistry, the level of tumor hypoxia using NMR spectroscopy, CD68, CD34, VEGF, and VEGFR-1 (Flt-1) expression using immunohistochemistry, and MMP-2 and MMP-9 activity using zymography. *Results. *DTCs were detected in 51.4% of GC patients with M_0_. There was significant correlation between frequency of DTCs in BM and level of tumor hypoxia (*P* < 0.024). DTCs presence was accompanied with Flt-1 positivity of BM. The correlation between DTCs and tumor VEGF expression in patients with M_0_ was shown (*P* < 0.0248). Activity of MMP-2 and MMP-9 in BM was linked with DTCs in patients with M_0 _ (*P* < 0.05). Overall survival (OS) of patients with M_0 _ and DTCs was shorter than that of patients without DTCs (patients in both groups were operated only) (*P* = 0.0497). *Conclusion. *Appearance of DTCs correlates with hypoxia level in primary tumors. Detection of DTCs in GC patients may be relevant indicator for adjuvant chemotherapy using.

## 1. Introduction

Gastric cancer is one of the most common cancers in Europe ranking the fifth after lung, prostate, colorectal, and bladder cancers in men and breast, colorectal, lung, and cancer of the corpus uteri in women [1]. In Ukraine in 2011 the annual age-standardized incidence rate was 21.5/100.000 ranking the fourth after lung, skin, and prostate cancers in men and 8.8/100 000 ranking the seventh after breast, skin, corpus uteri, colon, cervix uteri, and rectal cancers in women [2]. The therapy outcome of gastric cancer is still not satisfactory, and distant metastasis is kept as a key factor in the unfavorable results of gastric cancer treatment. It is known that even curative resection can not always guaranty expected long-term results of therapy that may be explained by the early dissemination of tumor cells even still before the surgical intervention [3–5]. It is shown that “tumor cells can disseminate from the earliest preneoplastic lesions, sometimes even before the formation of overt primary tumors” [6]. At the same time it is unable to detect early metastasis up till now despite modern tools, for example, MRT, PET.

Tumor cells leaving primary site can mainly settle in bone marrow (BM) with potency to form the metastases. These cells named disseminated tumor cells (DTCs) cannot be detected by conventional cytological methods, but they may be found both by immunocytochemistry and molecular technologies.

It was shown that DTCs in BM may be detected in 25–60% of patients with different tumors categorized as M_0_ [4–7]. Conception of the detection of micrometastases in BM has been introduced in clinical practice more than 30 years ago [8]. BM is considered now as a common homing-organ for DTCs that escape from epithelial tumor, first of all from breast, lung, prostate, and colorectal cancers. It was shown that persistent DTCs in BM are associated with unfavorable prognosis for breast cancer patients [9].

Perhaps firstly publication concerning DTCs in gastric cancer patients appeared in 1991 when Schlimok et al. [10] have shown the presence of DTCs in BM of 30% patients with gastric cancer without obvious distant metastases. The correlation between DTCs and some clinicopathological characteristics, in particular regional and distant metastasis, and Lauren classification was also determined. Further studies confirmed these data and focused one's attention on methodology of DTCs search and pointed out the clinical relevance of DTCs in gastric cancer patients, in particular their negative impact on survival [11–17]. Kolodziejczyk et al. [18] studying the influence of neoadjuvant chemotherapy on DTCs in gastric cancer patients have shown that frequency of DTCs finding was significantly decreased after chemotherapy but without substantial impact on disease outcome. Nevertheless, authors proposed to continue these studies, in particular in multicenter protocol investigations. At the same time some observations have been published where the clinical relevance of DTCs in gastric cancer patients was not observed [19–21]. Recently published review of Bidard et al. [22] allows to conclude that problem of DTCs in gastric cancer is relevant for clinics but it is not fully solved and needs to be clarified to improve the treatment outcome.

Our study was aimed to evaluate the influence of DTCs in BM of gastric cancer patients on survival and their prognostic significance. Moreover, our attention was focused on the assessment of possible correlation between DTCs in BM and hypoxia profile in primary tumor exploiting well-known fact that solid tumors are hypoxic that mediates tumor aggressiveness and poor disease outcome [23].

## 2. Patients and Methods

### 2.1. Patients

A total of 89 patients (62 men and 27 women) with primary gastric cancer (GC) were diagnosed and treated at the City Clinical Oncological Center (Kiev), during period 2008–2011 (Table 1). No patient received chemotherapy or radiation prior to surgery. Tissue samples were taken immediately after tumor excision. Tumors were classified and staged according to the 2002 version of the UICC staging system [24]. Histological types of tumor were evaluated by WHO histological classification (2000) [25]. All patients were thoroughly informed about the study that was approved by the local ethics committee.

### 2.2. Detection of Tumor Cells in Bone Marrow

Preoperatively, 2.0-3.0 mL of BM aspirates from the sternum with conventional cautions to avoid the hit of skin epithelial cells into the sample was taken into a heparinized syringe and transferred into a tube “Sarstedt” containing EDTA K. After Ficoll-Hypaque density centrifugation (density, 1.077; Sigma-Aldrich, USA) to isolate the mononuclear cell fraction (1105 g for 20 minutes), the interphase was washed twice in phosphate-buffered saline (PBS) with removing of erythrocytes (Uti-Lyse Erythrocyte Lysing Reagent, Dako Cytomation, USA), resuspended to a concentration of 570 · 10^3^ cells/30 *μ*L, and cytocentrifuged on glass slides. Specimens were air-dried from 12 to 24 hours and stained immediately or stored at –20°C.

Detection of tumor cells (cytokeratin-positive cells, CK-positive cells) in BM cytospin preparations fixed in acetone was provided by APAAP method (alkaline phosphatase-antialkaline phosphatase) and visualization system EnVision G/2 System/AP Rabbit/Mouse (Permanent Red) (Dako Cytomaiton, Denmark). Monoclonal mouse antibodies against panCK (clone AE1/AE3, Dako Cytomation, Denmark) were used as primary antibodies. Each assay was controlled negatively by staining of one cytospin preparation with nonspecific IgG_1_ (MOPC21, Sigma). Number of tumor cells (CK-positive cells) was expressed on 10^6^ BM mononuclear cells. BM samples were scored “positive” if the presence of two or more CK-positive cells per 10^6^ mononuclear cells was detected (from 6 to 12 slides per patient were screened).

### 2.3. Detection of Flt-1-Positive Cells in Bone Marrow with Immunohistochemical Method

Cytospins were fixed by formol-acetone solution (pH 6.6) in accordance with the instruction. Slides were treated by 0.3% Triton X-100 solution, washed by PBS and blocking of endogenous peroxidase followed by incubation in 3% bovine serum albumin to switch off nonspecific reaction antigen-antibody. Cytospins were incubated with primary polyclonal rabbit antibodies against Flt-1 (sc-316, Santa Cruz Biotechnology, Inc., USA) in optimal dilution 1 : 80 within 1 h. After washing of primary antibodies slides were processed with PolyVueHRP Detection System Components (Diagnostic BioSystems, USA).

### 2.4. Immunohistochemical Examination of Tumor Tissue

Expression of CD34 (the endothelial cell marker), CD68 (the commonly used macrophage marker), and VEGF was evaluated on deparaffinized slides by means of immunohistochemical staining using specific monoclonal mouse antibodies: clone QBEnd 10 (1 : 100), clone PG-M1 (1 : 80), and clone VG1 (1 : 50), respectively. Immunoreactions were detected and visualized with the polymer-peroxidase method (EnVision+/HRP and 3,3-diaminobenzidine; Dako Cytomation, Denmark) followed by counterstaining with Mayer hematoxylin. Positive controls were used as monoclonal antibodies against cytokeratins (clone MNF116, DakoCytomation, Denmark). Nonimmunized serum or PBS was substituted by primary antibodies as the negative control.

Microvessel density (MVD), detected by immunostaining for CD34, was assessed by the hot spot method [26]. CD68-positive cells were counted per 1000 cells in each slide and the number of CD68-positive cells was reported as percent. VEGF expression was assessed by scoring the number of all positive cells per 200x field. When the tumor consisted of less than 25% immunoreactive cells, the case was scored as weak, cases with 26–50% immunoreactive cells were scored as moderate, and those with 51–100% immunoreactive cells were scored as strong.

Expression of VEGFR-1 (Flt-1) was detected using polyclonal rabbit antibodies against Flt-1 (sc-316 (1 : 80), Santa Cruz Biotechnology, Inc., USA). Normal rabbit IgG (Dako Cytomation, Denmark) was used for the negative control. When the tumor consisted of more than 10% immunoreactive cells, the case was scored as positive.

### 2.5. Metalloproteinase-2 and Metalloproteinase-9 Activity Assay

Tumor specimens and BM aspirates (0.5 mL) were placed into the liquid nitrogen up to processing. Activity of MMP-2 and MMP-9 was determined for each sample by zymography in 12% polyacrylamide gel with SDS and 0.1% of gelatin as substrate [27].

### 2.6. ^31^P NMR Spectroscopy

Level of tumor hypoxia was assessed with ^31^P NMR spectroscopy. ^31^P NMR spectra of perchloric acid (PCA) tumor extracts were acquired by means of a high-resolution Bruker 400 MHz spectrometer (Widebore Ultrashield, AV-400 electronics, Germany) using a probe of 5 mm inner diameter. All details of method were presented in our earlier publication [27].

### 2.7. Statistical Analysis

All statistical analyses were conducted using the NCSS 2000/PASS 2000 and Prism, version 4.0 software packages. Correlations were analyzed with the Pearson correlation coefficient. The survival proportion was estimated by using the Kaplan-Meier method and differences in survival were analyzed with the log-rank test. Prognostic values of relevant variables were analyzed by means of the Cox proportional hazards model using hazard ratio and *χ* ^2^ test. Two-tailed *P* values <0.05 were considered statistically significant.

## 3. Results

### 3.1. Tumor Cells in Bone Marrow and Their Correlation with Clinical Variables

Individual patient data from a total of 89 histologically confirmed gastric cancer patients were included in this study (Table 1). The median age was 62 years. Overall, 51 patients (57.3%) had DTCs during follow-up. The mean number of DTCs in BM was 5.5 ± 1.0/10^6^ mononuclear cells. Patients with category M_0_ had DTCs in BM in 51.4% of cases (6.5 ± 2.4 CK-positive cells per 10^6^ mononuclear cells), and patients with category M_1_ in 78.9% cases (8.0 ± 2.0 CK-positive cells per 10^6^ mononuclear cells). There was no association of DTCs in BM with clinicopathological characteristics (Table 2). Meanwhile, the association between the presence of CK-positive cells in BM and level of hypoxia in primary tumor was detected: severe and moderate hypoxia was found in 75% of primary tumors in patients with DTCs in BM while mild and weak hypoxia in 32.2% only (*P* < 0.01). Level of tumor hypoxia assessed by NMR spectroscopy [27] was ranged as follows: if the PME/Pi < 1.0, tumors are characterized by severe hypoxia, 1.0 < PME/Pi < 1.4 moderate hypoxia, 1.4 < PME/Pi < 2.0 mild hypoxia, and PME/Pi > 2.0 weak hypoxia (satisfactory oxygenation).

It was also determined that the probability of appearance of tumor cells in BM of patients with category M_0_ is increased by a factor of 11.4 (odds ratio 11.4, 95% CI 2.71–47.89, *χ* ^2^ = 12.3, *P* < 0.001) when tumors were characterized by severe and moderate hypoxia.

### 3.2. Flt-1-Positive Cells in Bone Marrow and Primary Tumor

It was found that Flt-1 positive cells were detected both in BM and tumor, in 58.5% and 79% of patients, respectively. The presence of CK-positive cells in BM was accompanied with the Flt-1-positivity of BM in 67% and the absence of CK-positive cells with the Flt-1-positivity in 45% of cases. The correlation between the number of Flt-1-positive tumor and Flt-1-positive BM was not observed. The probability of the presence of Flt-1-positive cells in BM was increased by a factor of 2.7 when tumors were characterized by severe and moderate hypoxia although this probability was not statistically significant (odds ratio = 2.7; 95% CI 1.76–4.72; *P* > 0.05). The mean number of Flt-1-positive cells in tumor was 34 ± 3.0% (median 47%, range 0–96).

### 3.3. VEGF-Positive Cells, CD68, and MVD in Primary Tumor and CK-Positive Cells in Bone Marrow

Positive reaction with monoclonal antibody to VEGF was observed in 73% of tumors. It was shown that CK-positive cells in BM were detected in 73% of patients with VEGF-positive tumors. The direct correlation between VEGF-positive cell number in tumor and DTCs in BM was observed (*r* = 0.542; *P* < 0.025). The tendency was only assessed for the correlation between CK-positive BM and number of CD-68-positive cells as well as MVD in primary tumor (*P* > 0.05).

### 3.4. Activity of Gelatinases in Primary Tumor and Bone Marrow and CK-Positive Cells in Bone Marrow

It was shown that the association between activity of MMP-2 in tumor and presence of DTCs in BM, in particular MMP-2 activity was 9.2 ± 5.1 *μ*g/g tissue in patients with DTCs in BM whereas MMP-2 activity, was 4.1 ± 2.8 *μ*g/g in patients without DTCs in BM (*P* < 0.05). The association of tumor MMP-9 activity with DTCs in BM was not found. At the same time activity of both gelatinases in BM was linked with DTCs in BM; in particular activities of MMP-2 and MMP-9 were 8.6 ± 4.0 and 7.5 ± 3.4 *μ*g/g in patients with DTCs in BM and 2.8 ± 1.4 and 2.6 ± 1.85 *μ*g/g in patients without DTCs in BM (*P* < 0.05 and *P* < 0.05, resp.). It has to be noted that patients with category M_0_ were analyzed only.

### 3.5. Overall Survival of Patients with and without Tumor Cells in Bone Marrow and Treated and Not Treated with Adjuvant Chemotherapy

**Figure 1:**
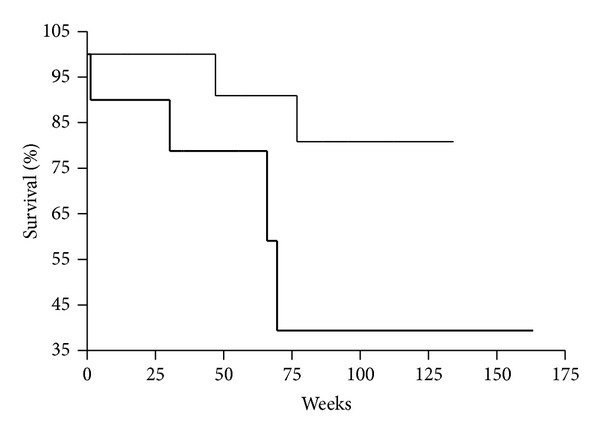
Kaplan-Meier overall survival curves for gastric cancer patients as a function of DTCs presence in bone marrow (DTCs^−^, thin line; DTCs^+^, bold line; *P* < 0.0497). Patients with M_0_ category were analyzed and operated only.

Overall survival (OS) of patients with category M_0_ with DTCs in BM was shorter than that of patients with category M_0_ and without DTCs in BM (*P* = 0.0497) (Figure 1). It has to be noted that patients in both groups were operated only. It can indicate that the detection of DTCs in BM may be considered as obligatory procedure before the decision concerning further treatment, in particular of patients with category M_0_.

Median follow-up time was 19.4 (range, 2.6–60.85) months from diagnosis for all patients (mean 21.9 ± 1.9). OS was significantly shorter in patients with M_0_ category and with DTCs, compared with patients with no DTCs (log-rank test: *P* < 0.05). If overall survival was analyzed for all patients (M_0_ and M_1_ categories) the difference was not statistically significant (*P* > 0.05).

Overall, 30 patients (33.7%) died during follow-up. In 26 patients (86.7%) death was related to gastric cancer. Of these, 20 patients (66.7%) had DTCs in BM. Of 20 patients with DTCs in BM, 8 patients (40%) had M_0_ category. Of 26 patients, who died, 13 (50%) have received any kind of adjuvant chemotherapy, and 10 (38.5%) had DTCs in BM (4 patients (40%) had M_0_ category). Survival in these patients was significantly shorter in patients with M_0_ category with DTCs compared with those patients with no DTC: 22.5 versus 39.5 months (Student's test, *P* < 0.05). Survival in all patients (with M_0_ and M_1_ categories) was almost the same (22.2 and 24.9 months, resp.) independently from DTCs in BM.

**Figure 2:**
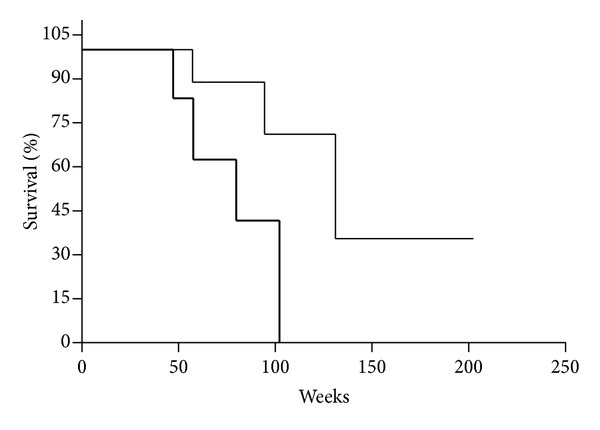
Kaplan-Meier overall survival curves for gastric cancer patients as a function of DTCs presence and Flt-1 expression in bone marrow (DTCs^−^/Flt-1^+^, thin line; DTCs^+^/Flt-1^+^, bold line; *P* = 0.0437). Patients with M_0_ and M_1_ categories were analyzed and treated with operation and adjuvant chemotherapy.

It was also found that OS of patients with DTCs in BM and Flt-1-positive cells in BM was significantly shorter than that of patients with no DTCs in BM, but with Flt-1-positive cells in BM (*P* = 0.0437) (Figure 2). Patients in both groups were categorized as M_0_ and M_1_ and treated with adjuvant chemotherapy. OS of patients with category M_0_ was not influenced by Flt-1 positivity of BM (*P* > 0.05).

**Figure 3:**
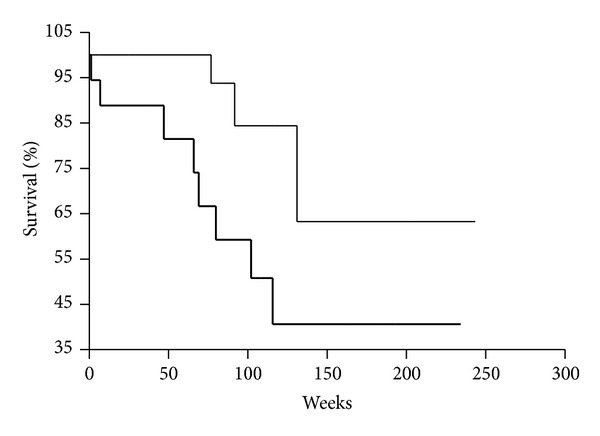
Kaplan-Meier overall survival curves for gastric cancer patients as a function of DTCs presence in bone marrow and VEGF expression in tumor (DTCs^−^/VEGF^+^, thin line; DTCs^+^/VEGF^+^, bold line; *P* < 0.0486). Patients with M_0_ and M_1_ categories were analyzed and treated with operation and adjuvant chemotherapy.

**Figure 4:**
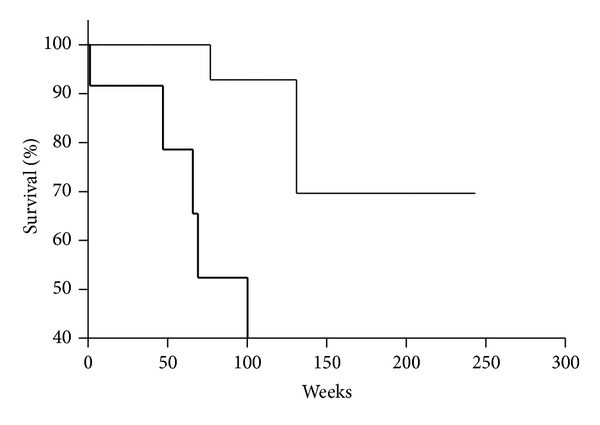
Kaplan-Meier overall survival curves for gastric cancer patients as a function of DTCs presence in bone marrow and VEGF expression in tumor (DTCs^−^/VEGF^+^, thin line; DTCs^+^/VEGF^+^, bold line; *P* = 0.0248). Patients with M_0_ category were analyzed and treated with operation and adjuvant chemotherapy.

Moreover, it was evaluated that OS of patients with DTCs in BM and VEGF-positive tumor was significantly shorter than that of patients without DTCs in BM and VEGF-positive tumor (*P* = 0.0486) (Figure 3). The patients in both groups were treated with adjuvant chemotherapy and diagnosed as categories M_0_ and M_1_. It is relevant that OS of patients with category M_0_ and DTCs in BM and VEGF-positive tumor was significantly shorter than that of patients with category M_0_ and without DTCs in BM and VEGF-positive tumor (*P* = 0.0248) (Figure 4). Patients in both groups were treated with adjuvant chemotherapy.

It was also found that in patients with category M_0_ but with DTCs in BM who have been operated only risk of unfavorable outcome increased by a factor of 2 (HR = 2.0; 95% CI = 0.98–5.76; *P* < 0.05). These data can indicate the necessity of the additional diagnostic procedures for patients with M_0_ established by conventional methods because some of them may have DTCs in BM and need of adjuvant therapy.

## 4. Discussion

Our study found that tumor cells present in BM of 51.4% patients with gastric cancer with category M_0_. DTCs in BM were detected in 35–60% of gastric cancer patients with category M_0_ by other authors [7]. It has to be noted that there are not enough publications concerning the significance of DTCs in BM in patients with M_0_. At the same time, namely, this information is more relevant than that in regard to patients with M_1_ that are diagnosed by conventional methods. It should be noted that association between DTCs in BM and the level of hypoxia in the primary tumor was found: tumor cells in BM were found in 80% of cases where severe hypoxia was found in the primary tumor, whereas under moderate and mild hypoxia DTCs they were found only in 20% of cases (*P* < 0.05). It was also determined that a probability of DTCs appearance in BM is increased by a factor of 11(odds ratio 11.8, 95% CI 3.0587–45.60, *χ* ^2^ = 14.79, *P* < 0.001) when primary tumors are characterized by severe hypoxia. These data indicate a possible positive impact of hypoxia-associated signaling pathways on the escape of tumor cells from the primary tumor and their dissemination into the BM to form a premetastatic niche as suggested by Kaplan et al. [28].

Close correlation was observed between DTCs and VEGF expression in primary tumor confirmed by early observation [15], but correlation between DTCs and tumor MVD was not found in contrast to other authors [14, 29]. In regard to Flt-1 expression in BM it has to be noted that our study confirmed data obtained by Mimori et al. [30] that simultaneous presence of tumor cells and Flt-1-positive cells in BM is clinically relevant for metastasis.

The role of MMPs in tumor dissemination is well known, but MMPs activity in BM of gastric cancer patients was not determined till now. Our study has shown that activity of MMP-2 and MMP-9 in BM of patients with M_0_ but with DTCs was increased by a factor of 3.1 and 2.9, respectively. It allows suggesting that gelatinases may play a significant role in the formation of premetastatic niche, in particular, in the reorganization of cellular microenvironment in BM.

In conclusion, it may be summarized that 51.4% of gastric cancer patients categorized as M_0_ have tumor cells in BM. Presence of DTCs is correlated with level of tumor hypoxia and accompanied with Flt-1 positivity of BM. The probability of Flt-1 positivity of BM was increased by a factor of 2.7 in patients with severe and moderate hypoxia in tumor. The significant correlation between CK-positivity of BM and VEGF expression as well as MMP-2 activity in tumor was shown. There is relevant observation that activity of both gelatinases in BM correlated with presence of DTCs in BM of patients with M_0_ only. OS of patients with M_0_ with DTCs in BM was significantly shorter than that in patients without DTCs. It has to be noted that patients in both groups were operated only. It was also evaluated that OS of patients with DTCs and Flt-1 positivity of BM was significantly shorter than that of patients without DTCs but with Flt-1-positive BM (these patients were treated with adjuvant chemotherapy). Detection of DTCs in gastric cancer patients may be as relevant indicator for personalised cancer therapy, in particular in the choice of treatment tactic for GC patients, especially with category M_0_. Perhaps, DTCs in BM may be as an argument to use adjuvant chemotherapy, though some other factors have to be taking into account, for instance, the influence of hostile microenvironment on DTCs dormancy in BM.

## Figures and Tables

**Table 1:** Patient and tumor characteristics.

Characteristics	Number, 89 (%)
Gender	
Male	62 (69.7)
Female	27 (30.3)
Age (years)	
Median	62
Range	34–84
Tumor location	
Upper third	9 (10.1)
Middle third	29 (32.6)
Lower third	47 (52.8)
Total	4 (4.5)
UICC stage	
I	22 (24.7)
II	16 (18.0)
III	23 (25.8)
IV	28 (31.5)
Histological type	
Adenocarcinoma	61 (68.5)
Mucinous adenocarcinoma	12 (13.5)
Signet-ring cell carcinoma	13 (14.6)
Undifferentiated carcinoma	3 (3.4)
Grade (G)	
1	6 (6.7)
2	15 (16.9)
3	60 (67.4)
4	8 (9.0)
T-classification	
T_1_	10 (11.2)
T_2_	18 (20.2)
T_3_	33 (37.1)
T_4_	28 (31.5)
Nodal involvement	
N_0_	43 (48.3)
N_1_	17 (19.1)
N_2_	29 (32.6)
Distant metastasis	
M_0_	70 (78.6)
M_1_	19 (21.4)

**Table 2:** Prevalence of disseminated tumor cells in bone marrow by clinical variables.

Variables	All patients (*n* = 89, 100%)	Patients with DTC (*n* = 51, 57.3%)	Patients without DTC (*n* = 38, 42.7%)
Gender			
Male	62	27 (43.5)	35 (56.5)
Female	27	17 (63.0)	10 (37.0)
Patients age groups (*n*, %)			
≤65	56	35 (62.5)	21 (37.5)
>65	33	16 (48.5)	17 (51.5)
Age (years; median, range)	62 (34–84)		
Tumor location			
Upper third	9	6 (66.7)	3 (33.3)
Middle third	29	13 (44.8)	16 (55.2)
Lower third	47	22 (46.8)	25 (63.2)
Total	4	3 (75.0)	1 (25.0)
UICC Stage			
I	22	11 (50.0)	11 (50.0)
II	16	6 (37.5)	10 (62.5)
III	23	10 (43.5)	13 (56.5)
IV	28	17 (60.7)	11 (39.3)
Histological type			
Adenocarcinoma	61	26 (42.6)	35 (57.4)
Mucinous adenocarcinoma	12	8 (66.7)	4 (33.3)
Signet-ring carcinoma	13	8 (61.5)	5 (38.5)
Undifferentiated carcinoma	3	2 (66.7)	1 (33.3)
Grade (G)			
1	6	3 (50.0)	3 (50.0)
2	15	4 (26.7)	11 (73.3)
3	60	33 (55.0)	27 (45.0)
4	8	4 (50.0)	4 (50.0)
T-classification			
T_1_	10	3 (30.0)	7 (70.0)
T_2_	18	13 (72.2)	5 (27.8)
T_3_	33	15 (45.6)	18 (54.5)
T_4_	28	13 (46.4)	15 (53.6)
Nodal involvement			
N_0_	43	18 (41.9)	25 (58.1)
N_1_	17	9 (52.9)	8 (47.1)
N_2_	29	17 (58.6)	12 (41.4)
Distant metastasis			
M_0_	70	36 (51.4)	34 (48.6)
M_1_	19	15 (78.9)	4 (21.1)
Systemic therapy			
Operation only	47	27 (57.4)	20 (42.6)
Adjuvant chemotherapy	42	24 (57.1)	18 (42.9)
